# “Remember, we don't have race categories here”: contradictions and reflections on racism, environment, and health from an interview study among Black German researchers, educators, and care providers

**DOI:** 10.3389/fpubh.2025.1658436

**Published:** 2025-12-19

**Authors:** Devon C. Payne-Sturges

**Affiliations:** 1 Department of Environmental Health Sciences, University of Michigan School of Public Health, Ann Arbor, MI, United States; 2 Department of Global, Environmental, and Occupational Health, University of Maryland School of Public Health, College Park, MD, United States

**Keywords:** Germany, African diaspora, environmental justice, structural racism, qualitative research, environmental health, health equity, health monitoring

## Abstract

**Background:**

The global reckoning on race and racism in 2020 ushered in new or enhancement of existing governmental anti-discrimination and anti-racism initiatives at EU and German Federal levels. However, the role of racism, particularly structural racism, in health appears to be missing from community health and medical training and research, and from larger policy debates on environmental justice and health equity in Germany.

**Design/Methods:**

Participants were purposively recruited across 10 German cities selected to represent regional variability in racial diversity and engaged in semi-structured in-depth interviews that were audio-recorded and transcribed verbatim. Using thematic analysis, these qualitative interviews with 14 anti-racism researchers, community-based advocates and educators knowledgeable about environmental justice, racism, public health who identified as Black or Afro-German and/or work on behalf of racialized communities in Germany were analyzed to explore the challenges faced by minoritized communities in Germany in relation to environmental justice and health inequities, as well as strategies for addressing these issues.

**Results:**

Participants enumerated a number environmental conditions likely disproportionately impacting racialized minority groups in Germany. The extent of these issues is not known due to lack of data and empirical studies. Participants pointed to the myriad ways racism is structured in Germany, tracing how racialized and racially hierarchical values and beliefs become actualized through racially discriminatory policies and practices embedded in social institutions such as the government, the economy, the education system and the healthcare system which lead to racialized outcomes in health and environmental conditions.

**Conclusions:**

Interview participants identified pathways toward more effective research and policy initiatives on racism, environmental justice, community health and environmental heath equity in Germany including: collecting better data that is informed by structural theories of race/racialization and power; addressing history and national narratives; supporting more community-based participatory action research; engaging with existing civil society and non-governmental organizations that serve and advocate on behalf of minoritized communities; and building upon past and present progressive social movements.

## Introduction

Environmental conditions are major contributors to health and well-being, but they are not evenly distributed across population groups, shaping environmental health inequities in all European countries (1). Environmental health inequities are framed as related to socioeconomic, sociodemographic or spatial differences in exposure to environmental health risk factors and to differences in health status caused by environmental conditions (1). For example, data show that in many European countries, including Germany, lower socio-economic groups living in urban areas are disproportionately exposed to air pollution, noise and high temperatures (2). Risks of negative health impacts from these exposures, such as cardiovascular disease, neurodevelopmental disorders, adverse pregnancy outcomes, mental illness and premature mortality, may be heightened by factors such as limited or no access to healthcare and green space, poverty, and unemployment (2–5). The social, political and economic drivers of harmful environmental stressor exposures disproportionately impacting low-wealth and racialized communities have been the targets of the environmental justice movement in the U.S., which has inspired environmental justice research and advocacy efforts internationally (6–8). But in Germany, the public health monitoring of *sociodemographic* differences in environment and heath has focused narrowly on “migration background” or “migration history” as an indicator of social disadvantage, which is problematic. This approach ignores racism as a root cause of environmental and health inequities among racialized minority groups (9). People facing racial discrimination are not necessarily represented by the statistical category “migration background” (10–13) used in Germany for health monitoring. At the same time, the term “migration background” creates the perception of people put in this category as “strangers” or “others” even when they are born and raised in Germany and/or one of their parents is German. This contributes to racialization of certain populations and communities. In Germany there is greater focus on health effects of migration and less on racism as an independent causal factor.

Yet, the historic global reckoning on race and racism in 2020 brought to the forefront evidence of police violence against racialized minorities, race-based discrimination in housing, employment and education, and racial disparities in the impact of COVID-19 across Europe and even in Germany (14–24). Health and social justice scholars and activists have argued that the same processes that led to disproportionate killings of unarmed Black men by police are also linked to the racial/ethnic disparities in COVID-19 and environmental injustice (3, 22, 25–32). Health researchers have been inspired to move away from merely describing racial disparities and move toward investigating the institutions and systems that create inequities (33–35). Each One Teach One, a community-based organization headquartered in Berlin, conducted the groundbreaking Afrozensus survey of over 6,000 Black, African and Afro-diasporic people in Germany in 2019–2020 and found that 6 out of 10 respondents (64.6% of *n* = 3,385) who had contact with the health care sector in the last two years stated that they had experienced discrimination there (14). The events of 2020 also created urgency for enhancing existing or ushered in new governmental anti-discrimination and anti-racism initiatives at EU (36) and German Federal levels (37–39). Since the Afrozensus, a number of German governmental reports have been published including *Rassismus und seine Symptome* (Racism and Its Symptoms) (16) and *Rassismus Forschung I Theoretische und interdisziplinäre Perspektiven* (Racism research I Theoretical and Interdisciplinary Perspectives) (40) both published by the German Center for Integration and Migration Research (DeZIM Institute), the EU level report, Being Black in the EU: Experience of People of Africa Descent (41) and *Den alltäglichen und den institutionellen Rassismus bekämpfen!* (Parallel report to the UN Committee for the Elimination of Racial Discrimination (ICERD) to the 23rd–26th Report of the Federal Republic of Germany) (42). These reports were published just in 2023 and basically corroborated the community-led Afrozensus. However, the role of racism, particularly structural racism, in health appears to be missing from community health and medical training and research, and from larger policy debates on environmental justice and health equity in Germany. If racism is indeed on the political agenda in Germany, why is there a disconnect when it comes to health and the environment? What are the solutions?

This article aims to address these questions from the perspectives of environmental justice, anti-racism, public health and environmental justice researchers, community-based advocates and educators who identify as Black or Afro-German and/or work on behalf of racialized communities in Germany. There is limited exploration on how racism is conceptualized regarding links between environment and health in the German context by activists and researchers of color. For the German public health sector (researchers, practitioners and government administration alike) to more effectively address racial inequities, it is essential to develop an understanding of the structures of racism and to hear directly from the people who are impacted. There are numerous groups organized to address racism (especially anti-Black racism), decolonialization movements, and scholars of race, ethnicity and politics but these groups are yet to be invited as partners shaping community health or environmental health research and administration in Germany. Therefore I undertook a qualitative study to investigate the perspectives of and insights into the challenges faced by minoritized communities in Germany (particularly Afro-German communities) in relation to environmental justice and health inequities, as well as strategies for addressing these issues and promoting positive change. I sought to address three main research questions.

1. What are examples of health and environmental inequities disproportionately affecting racially minoritized communities in Germany?
2. What are the historical, cultural and structural causal mechanisms for these inequities?
3. What are potential solutions for addressing racial inequities in health, healthcare and promoting environmental justice in Germany? What progress has been made, and what challenges remain?

The article is structured as follows. First, I provide a brief theoretical orientation to racism and its contribution to health and environmental health inequities. Second, I describe German governmental initiatives on racism since 2020 and point to disconnects vis a vis health and environmental justice. Third, I present my qualitative methodology and analysis of semi-structured interviews. I conclude with discussion of the themes from the interviews and recommendations.

## Contextual background

### Racism, health and environment

Racism can be globally defined as. interlocking systems of power and policy decisions “that unfairly disadvantage some individuals and communities, unfairly advantage other individuals and communities” based on racial hierarchy, and “sap the strength of the whole society through the waste of human resources” (43). Racism is linked to the creation of harmful living conditions and environmental inequities, including the disparate impacts of the climate crisis, through discriminatory practices of residential segregation, in education, employment, criminal justice and health care, and in exposures to toxic chemicals and disproportionate siting of polluting sources in racial and ethnic minority communities (3, 4, 44–48). Environmental exposure inequities (3, 49–53), which have negative consequences for health, have been identified as “a form of contemporary state-sponsored, slow racial violence tied to forms of historical racial violence through the common thread of cultural racism (i.e., a society's ideologies and value systems, or culture, based on implicit and explicit ideas of a racial hierarchy)” regarding whose lives are valued and whose are not (31). These environmental exposure inequities produced by racism become embodied (54), “under skin” of racialized groups and individuals, which we observe as the social patterning of population health outcomes (4, 44, 55–59). Since the beginning of the environmental justice movement in the U.S., protecting health and improving the quality of life of low income and racial minority communities have been critical objectives in the fight against environmental injustices, the ostensibly race-neutral policies or practices that result in racially unequal environmental exposures, whether intended or not (6, 7, 60).

**Figure 1:**
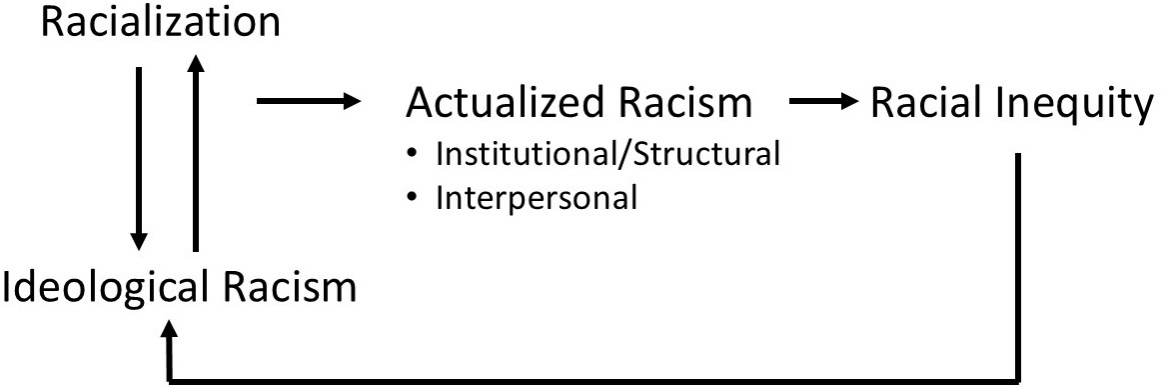
Conceptual model linking racialization to racial inequity via ideological and actualized racism. Adapted from (63).

While documenting or monitoring environmental health inequities is important, it is insufficient, to alleviate the disparities. An important step toward lasting environmental justice is specifying the underlying social mechanisms of racism (47, 61, 62) that initiate and perpetuate racial/ethnic differences in exposures to environmental hazards as plausible explanations for poor outcomes across the lifespan. Thus, improving the operationalization of racism in empirical public health research that can inform policy is needed. To this end Needham and colleagues offer a conceptual model linking cultural or ideological racism of racial superiority/inferiority (white supremacy), racialization, actualized racism (institutional/structural and interpersonal) to racialized inequities in health outcomes (63). Racialization is the social construction of racial categories, such as White, Black, Latino, Asian, Roma and even “migrant”. It's important to note that racialization of groups also occurs based on perceived cultural differences, using indicators like religion, clothing, nationality to attached negative stereotypes (31, 64, 65). Cultural/ideological racism and racialization reinforce each other and are not unique to the U.S. These processes can be identified and assessed by how they manifest, impacting different racialized groups in different ways, over time and place within and between countries (64). The actualization or application of cultural/ideological racism occurs at both institutional/structural and interpersonal levels. Needham defines institutional racism as racially discriminatory policies and practices embedded in social institutions such as the government, the economy, the education system, the healthcare system, religious institutions, the family, and the media (63). These policies and practices can appear neutral on the surface but actually produce or sustain racial inequities between racial/racialized groups. Further, institutional racism is said to be systemic or structural when it operates as a system across multiple interconnected institutions and policy decisions. Interpersonal racism refers to discriminatory treatment by race among individual actors (63). The results of these linked and reinforcing processes are the inequitable, or unjust, outcomes by race, including inequities in education, economic mobility, environmental conditions and health outcomes, which then are often used to justify racist ideology (e.g., scientific racism and eugenics). Thus, in racialized social systems such as the U.S. and Europe, the process of racial differentiation is inextricably intertwined with the process of racial stratification (i.e., the hierarchical ranking of people according to race/racialization), which results in differential access to power and other resources which affects the health and wellbeing of the population. The Needham framework (Figure 1) provides a helpful roadmap for tracing both the visible and invisible systemic and structural mechanisms of racism, and the impacts on health of different racialized groups, including environmental health inequities, in any context, including Germany.

### “Germany has a racism problem”: German initiatives on racism since 2020

Federal Minister for Family Affairs, Senior Citizens, Women, and Youth Franziska Giffey, declared that “Germany has a racism problem” at the first meeting of the *Kabinettausschuss zur Bekämpfung von Rechtsextremismus und Rassismus* or Cabinet Committee Against Right-Wing Extremism and Racism on May 20, 2020 (66). She was echoing earlier sentiment expressed by the then Federal Chancellor of Germany, Angela Merkel after the murders in Hanau (67, 68). The Cabinet Committee, newly created in March 2020, was in direct response to a string of high profile racially motivated violence in Germany that was attributed to perpetrators belonging to the extreme-right (69–71). In July 2020, the German Bundestag provided funding to establish National Discrimination and Racism Monitor (NaDiRa) and conduct research [Knowledge Network for Racism Research (WinRa) (72)] at the German Center for Integration and Migration Research (DeZIM Institute) and authorized funding for a 3 year study, “Racism as a Threat to Social Cohesion in the Context of Selected Socio-Institutional Areas” among other studies (73). Following public mobilization of the Black Lives Matter and anti-racism movements worldwide demanding racial justice after the violent death of George Floyd at the hands of the police, and longstanding calls from anti-racist organizations for EU action to tackle racism through a comprehensive strategy, the European Commission published an EU Anti-racism Action Plan 2020–2025 (36) in September 2020. Then the Cabinet Committee in November 2020 approved a €1 billion funding package to combat right-wing extremism, racism, anti-Semitism, anti-Muslim hostility in Germany and to establish a new National Anti-Racism Commissioner position in the Ministry of Migration, Refugees and Integration to coordinate the implementation of measures on protecting and increasing availably of community-based counseling for the victims of racism and right-wing extremist violence, addressing hate speech online, creating a network of advisory structures and preventing racism in sports (74). In January of 2023, the new National Anti-Racism Commissioner, Reem Alabali-Radovan, released the government's first-ever annual report on racism in Germany (75, 76), compiling data on racial violence, individual attitudes about race, and discrimination experiences from other organizations and reports such as NaDiRa, Berlin State Senate and the African-diaspora serving community-based organization Each One Teach One. A few months later in June 2023, Alabali-Radovan established an Expert Council on Anti-Racism consisting of 12 experts from science, administration and practice (though no one on the council has expertise in public health or environmental policy) to develop proposals for an effective anti-racism policy in Germany including creating a legal definition of racism (77).

### The disconnect and the denial

While the topic of racism has seen an increase in German public discourse (38, 40, 78–82), at the same time it is still considered taboo (20, 33, 83–85). Racism, and its cultural and structural causal mechanisms, have been omitted from discussions and governmental action with respect to environmental and climate justice and impacts on health in Germany (86, 87). As mentioned earlier, European research on environmental justice focuses almost exclusively on issues of income inequality and “migration background”. Furthermore, the environmental justice discourse in Western Europe is primarily taking place in elite spaces of academia, rather than being led by non-governmental organizations and civil society as in the US (8). “Racism, the dimension of oppression that shaped the US environmental justice movement in its early days, is largely absent from European studies” (86). Unlike in the U.S., sociodemographic data that allows for disaggregation by race and provides information on whether a person experiences racism is not generally collected in Europe by administrative bodies. In Germany this is very much the case. In postwar Germany, the terminology around *Rasse* and race was expunged from Germans' vocabulary and public discourse (84, 88). The term *Rasse* quickly became taboo (in German the word is unequivocally pejorative and also translates to “breed,” used for animals) and race was increasingly rejected as a key concept for thinking about difference within postwar German society (84, 88). Thus the German census only differentiates between Germans, Germans with “migration background” and foreigners. Without routinely collecting data on race and ethnicity, officials do not know exactly how many German residents and citizens are people of color or the full extent to which these populations face poorer health, social, and economic outcomes (42). As a result, there has been a paucity of empirical studies on racism in Germany, and community health and environmental researchers find studying racism “challenging” in Germany due to a European culture of racial denial (33, 83, 89–93) leaving them with a limited vocabulary to speak of racism. Naming racism a taboo topic belies reality however. The practice of racialized categorization is a daily occurrence embedded in the institutional practices and norms (20). “Migration background” is a proxy for race and has been interpreted as an indicator for cultural and even genetic differences in disease rates (19, 94–96). Further, race correction factors in algorithms to determine treatment in health care are used in Europe (97) without critical evaluation. German governmental anti-racism initiatives almost exclusively focus on individual victims of discrimination and visible acts of violence by racist actors all framed within a context of migration and integration. The danger then is that this could lead to policy proposals disconnected from structural causes, history and lived experiences of German citizens/residents of color who are not recent arrivals. This has implications for ensuring health equity and environmental justice in Germany, which will become clearer in the results and discussion sections.

## Methods

### Procedure

This study was conducted in Germany, from October 2023 to January 2024. I returned to Germany after spending time there in 2017 as a Fulbright Senior Specialist for environmental health and environmental justice research. At that time, I was invited to provide guidance and input on incorporating environmental health disparities and environmental justice topics into university level courses on community health because of my 20 years of experience in environmental health policy and environmental justice research in the U.S. During that time in 2017, I observed that environmental justice in Germany was mainly an academic topic dominated by public health and epidemiological research, and urban planning administrative agencies. Additionally, I learned that environmental justice as a topic in Germany had not been prompted by a social movement of affected groups (unlike in the U.S.) and that academics and governmental actors avoided using terms such as racial/ethnic discrimination or residential racial segregation because in their opinion these problems did not exist in Germany as they do in the U.S. This raised many interesting questions to pursue. For this initial study, adult community-based activists, Afro-German/Black German thought-leaders, environmental justice and anti-racism (including decolonization) activists and university-based and independent researchers were identified purposefully and recruited for interviews. Participants knowledgeable about environmental justice, racism, public health were recruited using targeted outreach and referrals from key informants and contacts from my prior visit across 10 German cities selected to represent regional variability in racial diversity. Participation was voluntary and participants were assured of confidentiality. After obtaining consent, semi-structured interviews of participants were conducted in English, either in person or on zoom, and lasted 90–120 min. For development of the interview guide, I relied on Needham's model and similar conceptualizations of racism as the macro-level systems, social forces, institutions, ideologies (e.g., white supremacy, zero-sum mentality), and processes that interact with one another to generate and reinforce racial/ethnic inequities (34, 44, 62, 63, 65, 98, 99). Interview questions thus focused on exploring instances of racial disparities in health and environmental conditions in Germany; perspectives on the institutional, structural and historical causes of these disparities; policy needs and possible solutions; relationships between various stakeholders working on environmental health issues; and opportunities for collaboration to reduce environmental and health inequities. All interviews were audio-recorded and anonymized transcripts were used for analysis. Self-reported demographic data were also collected. Additionally, I visited historical and cultural sites relevant to Afro-German community and postcolonial movements. Field notes and summary reports were written after each interview and site visit. IRB ethics approval was obtained from the University of Maryland (my academic institution at the time of the study).

### Analysis

I used analytic methods based on a reflexive thematic analysis approach (100, 101). This approach allows for a nuanced and flexible examination of the data, enabling the identification and interpretation of patterns and themes. My analysis followed six recursive phases: familiarization with the data, generating initial codes, searching for themes, reviewing themes, defining and naming themes, and producing the final report. Data familiarization involved repeated readings of the transcripts to immerse myself in the content. I took notes on the data as I reviewed the transcripts. Initial codes were generated inductively, capturing significant features across the dataset using NVivo 14 software (102). I then developed themes under each research question through iterative analysis, ensuring they were coherent and meaningfully distinct. The themes were reviewed and refined to ensure they accurately represented the data. Throughout the process, reflexivity was maintained by regularly reflecting on my own perspectives and potential biases, ensuring a rigorous analysis to answer my three research questions. The final themes were defined and named, capturing the essence of the patterns identified, and were supported by representative quotes from the data.

## Results

### Participant characteristics

Fourteen participants from seven German cities completed interviews. Majority self-identified as female (n = 12) and Black (n = 7). Others described themselves as Afro-German, Black Eastern German/POC, White/Turkish and White German. Most were professionally employed with either non-governmental organizations serving racialize communities, health care organizations, German Federal Government, private business, universities or were self-employed. Several were concurrently PhD students whose research focused on environmental justice, racism or health disparities. The characteristics of participants are shown in Supplemental information (Supplementary Table S1).

### Themes

**Figure 2:**
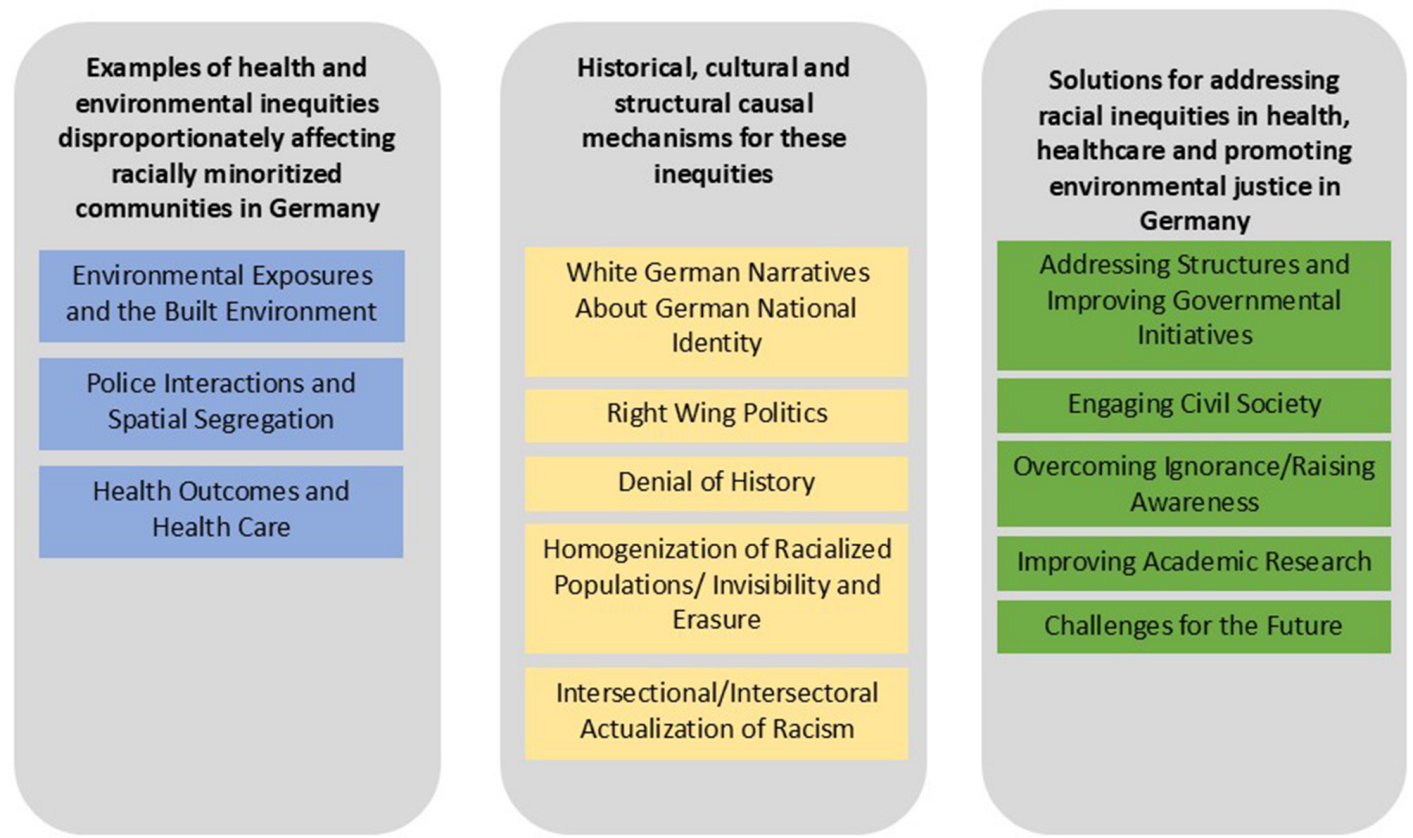
Themes.

Several themes were identified based on interviewee responses under each research question as shown in Figure 2. Examples of health and environmental inequities were grouped into three thematic areas of environmental exposures and the built environment, police interactions and spatial segregation, health outcomes and health care. When asked about causal mechanisms for environmental health inequities, participants provided many responses grounded in their lived-experiences, observations and in some cases their own scholarly research which were grouped into five themes including national narratives about German identity that erases certain populations, the growth in right-wing politics, denial of history, homogenization of racialized populations and intersectional/intersectoral actualization of racism. Lastly questions on solution spaces generated five themes that mostly dealt with addressing structures and improving governmental initiatives, engaging civil society, raising public awareness and improving research along with acknowledging political challenges. Detailed descriptions of the meanings I ascribe to each theme and the strength of endorsement by participant data are provided in Supplement information (Supplementary Tables S2–S4).

#### Environmental and health inequities disproportionately affecting racially minoritized communities in Germany

##### Environmental exposures and the built environment

Participants described a range of environmental exposures and stressors (e.g. air pollution, climate, flooding), work conditions (e.g., mining) and spatial segregation (e.g., lack of access to green space, concentrating African migrants and refugees in certain areas, pushing Roma community to the periphery of cities where there is pollution) that negatively impact the health of minoritized populations in Germany. Specific references were made regarding low housing quality for immigrants, Roma and Sinti communities, and other racialized people as well as segregation of housing and exploitation by landlords.

“Apart from being also in more heavily polluted areas, a lot of times the sanitation doesn't work, the electricity is not properly wired, and really like hazardous places where the state fails to really have the state guidelines and regulations like fully implemented.”—Participant 12, a PhD student and environmental justice activist in Berlin.

Participants acknowledged the general lack of governmental data on environmental conditions, health and race due to the avoidance by governmental agencies to consider racism as a causal factor. As a one participant from an NGO in Berlin (Participant 13) commented, the lack of data means it is not possible to evaluate health impacts of racism in the German context or to document racial health disparities. Instead one must rely on community knowledge to make these connections, which can be readily apparent as explained by Participant 12.

“Without knowing or having any official data where exactly most Black people live in Berlin but based on what is community knowledge, where we will find each other, we can deduct from the state (Berlin Environmental Justice Atlas on environmental pollution) that Black people in Berlin are disproportionately affected by environmental issues…pollution.”—Participant 12

##### Police interactions and spatial segregation

Discrimination by police was mentioned as an example of health and environmental inequity. Participant 12 linked heightened surveillance (racial profiling) of Black men and Muslim communities in Germany to environmental injustice in that this limits people' s access to certain spaces and enforces spatial segregation.

“For example discrimination that Black people experience while spending time in the park and using train/transport…I don't know if it's the same in the U.S. but here all sorts of stereotypes, specifically Black men being harassed by the police, or like suspicions of drug possession or drug dealing.”—Participant 12

Raising the topic of police interactions led to discussions about the impact of the global Black Lives Matter movement. Majority of interviewees pointed to the events of early 2020 as a turning point. Suddenly racism was part of German public discourse, including connections to health.

“So there were two things [murders in Halle and Hanau by right-wing sympathizers], plus the murder of George Floyd and the Black Lives Matter [movements and protests]. And it was the pandemic, so everything was quite quiet..and then suddenly it just sparked a political discussion.” -Participant 7, researcher in Berlin

Some participants pointed to a number of recently published reports on discrimination in daily life, including interactions with the police, and “everyday racism”, such as the groundbreaking Afrozensus survey of over 6,000 Black, African and Afro-diasporic people in Germany (14). One participant remarked that the EU level report, Being Black in the EU: Experience of People of Africa Descent (41) provided important overall context and international pressure for German society to address racism, especially in the wake of George Floyd protests.

“We are actually the most vulnerable of people in the world and in Germany, especially because according to the study, the recent study that came out, they said that Germany is the top in the European Union for racism against Blacks.”—Participant 4, community-based NGO in Köln

##### Health outcomes and health care

Participants spoke about racial disparities in specific health outcomes that can be linked both to environmental and social conditions such as COVID and mental health. They also described barriers to health care access and prevailing racist ideas about health behaviors of certain population groups. Similar to the experience in the U.S., the COVID pandemic revealed the depth of social inequities in Germany, racial ideology and misconceptions about race and genetics despite that research has shown the importance of living environments, especially how crowded housing and poor ventilation contribute to the transmission of SARS-CoV-2 and other infectious respiratory viruses between residents (103–106). Preventive measures recommended to the general public to reduce disease risk however are structurally constrained where racialized communities such as Black immigrants or Turkish people may live in segregated communities with substandard and crowded housing conditions, unsafe or limited water that make hand hygiene and social distancing challenging, if not impossible (29, 57). When describing how rich areas in the city Köln of had low incidence of COVID compared to higher incidence in areas with persons with “migrant backgrounds”, Participant 1 commented on popular arguments that were made during the early period of the pandemic to explain away the disparities.

“It was like ‘they are not so careful and this has to do with their culture they are not taking COVID serious enough'.. though this argument is not genetic but [it is] still cloaked in the in this “social/cultural” things [and] is replacement for racist arguments.”—Participant 1, community educator with NGO in Düsseldorf

Similarly Participant 11, a university professor of Turkish heritage shared that

“…the question I was mostly asked was why do Turkish people or people with a so called migration background, are much more affected by COVID than others? Is it about their neglecting the measures that have been taken to fight COVID? And then I, of course, explained very calm that, this is all about social background, about education and how the system approaches the people. So we formulated a statement against these arguments on migration and COVID and why we need solidarity.”—Participant 11, university professor

Environmental conditions can significantly impact mental health through exposure to neurotoxic chemicals and pollutants, psychosocial stressors (interpersonal racism), poor living conditions, and lack of access to green space (107–110). Disparities in mental health were mentioned often by participants. One participant who described himself as Afro-German explained how direct and indirect experiences of racism affect a person's “mindset” and become internalized.

“They have the feeling no matter what I am doing I am always doubted, I am not taken seriously if I want to be visible part to this society..people will never stop to ask me ‘where are you from' this gives people sometimes the feeling no matter what I am doing I am not seen as integrated in the society...[see me] as the other… you tend to underestimate yourself because of the narratives told about you”.—Participant 1

Self-doubt, trauma, stress, and environmental health conditions that could be addressed by a mental health care provider, are only compounded for Black persons or person of color or for those perceived as “of migration background” in Germany. The difficulty in obtaining referrals and finding a therapist, lack of mental health care providers who come from similar backgrounds as the minoritized patients, and white doctors' dismissiveness toward Black patients' symptoms of depression and anxiety due to psychosocial stressors such as experiences of interpersonal racism were all mentioned as reasons for worsening mental health outcomes among racialized persons.

“We're beginning to understand that racism impacts mental health and at the same time it's really hard to find a therapist. I think it's hard to find a therapist in general for most people and then if you add on top being part of a racialized group it just makes things even more difficult. So it's like that catch 22 of, you know, having a greater need, and at the same time having less.”—Participant 7

### Cultural, historical, structural causal mechanisms

#### White German narratives about German national identity

Modern scholarship on racism and health builds upon the evidence on the negative effects of interpersonal racism by advancing a structural model for conceptualizing racism or racialization, emphasizing the interaction of multiple institutions and individual actors in ongoing processes, animated by underlying values, beliefs and ideology of white supremacy, producing racialized outcomes (31, 62, 63). As explicated in Needham's model, ideological (and cultural) racism is “actualized” through ostensibly race-neutral policies and practices at institutional/structural levels as well as interactions at the interpersonal level (discriminatory treatment by race among individual actors). Majority of participants, from their lived experiences as parents, workers, students, and researchers, commented that a critical underlying issue is that German culture absolutely refuses to see itself as a country of diversity and pluralism.

“...there's still this idea in Germany that a real German is white. There's always an idea of, you have to be a foreigner [a person of color though born in Germany], you are different, you are like *Fremde*, you are alien to the society, you are not a real part of the society, and you will never be, because you are not white. In sociology, we would call the racism we have in Germany *Blut und Boden Racism* (blood and soil racism).”—Participant 13, freelance author/sociologist in Berlin

This narrative of leading or main [white] German culture also includes religion. Participant 11 shared that it is expected that residents of Germany should be familiar with the Christian religion, even if they do not practice it, and accept that German society is a Christian society. Political parties push this narrative which, according to Participant 11, contributes to the “neglect of the development of society” as a democracy and “fosters suspicion” of people who don't fit the image of a *real* German (especially Black people and Muslims) by saying that there is the German main culture and there is no migration transformation.

Directly related to the topic of environment and health, is the cultural narrative of Germanness as cleanliness. This has been invoked to draw lines of “us” and “the migrants.”

“In German culture, Germans see themselves as very clean and not polluting and, recycling. It does not go with the German self-imagination to have pollution, to produce pollution. So [if] there is pollution, it then must be the others. Like people were in an area where more migrants live, and instead of asking questions like, ‘how often is the trash picked up', it's always ‘people are dirty, people don't throw away their trash, people don't recycle.' ”—Participant 12

#### Right-wing politics

Participants noted how national identity narratives relate to right-wing politics and contribute to the growth in right-wing parties co-opting the environmental movement.

“The far right is also claiming more environmental even starting to claim climate issues for themselves…more in the sense of protecting the homeland (from foreigners).”—Participant 12

#### Denial of history

White or main German culture narratives are also related to how national history is retold or ignored. Majority of participants pointed to the many ways German society refuses to accept and actually reconcile with the country's colonial past which feeds a certain narrative to this day that with the defeat of the Nazis and the founding of a reunited Germany, racism doesn't exist.

“I think here in Germany, it's really a blind spot. Germans have difficulties to accept their colonial history, especially in the African countries. They always minimize it. World War II is a big topic, but not the colonial history from Germany.”—Participant 8, manager of an NGO in Berlin

“Once you start talking about colonialism and what they do is to compare and say the French colonialism was longer and so on. That's what we do in Germany. It [France's colonialism] was long and it was more brutal. ‘No, we did not do anything'.”—Participant 2, historian in Hamburg on post-colonialism

There are many decolonial initiatives (111–113) underway in Germany to educate people on topics such as the Berlin-Africa Conference (114) and the genocide of the Herero and Nama people (in what is present-day Namibia) committed by Germany under Bismark nearly four decades before the holocaust committed by Nazi Germany (115–117). But as several participants noted, the “blind spot” starts with how children and taught.

“Children are not taught history—doesn't matter if the person is 6 or 66.. about colonial past of Germany. They say it was short and has no impact on the present. This is the narrative. Germany was not like France or UK. [People] are of often very surprise to hear about the actual history.”—Participant 1

As one participant continues, this active denial, which begins with children's early education, allows German society to assuage collective guilt for WWII so they can just move on without real critical analysis.

“So at school the history of this country…[it] is like ok the Nazi regime during the second world war was really terrible so don't be anti-Semitic. That is the whole educational thing. And then barely no schooling or education on colonialism or colonial history… there is a lot of denial [of] German history and focus a lot on anti-Semitism. And they.. also like the government says ‘yeah maybe there is racism but we are busy with anti-Semitism'. Like ‘we don't have no other space to take care of anything else' or ‘Yeah we are reflecting on hour history'. [But] it is incomplete. It is also even the anti-Semitism reflection or teaching is not.. they don't learn what anti-Semitism is..they just learn about how bad the history was and that they shouldn't repeat it again.”—Participant 3, Community Activist and PhD student in Berlin

Participant 13, whose research focusses on the history of Black East Germans, noted that the narrative around WWII also undergirds scapegoating White East Germans for racism.

“'We abolish that [racism] with the defeat of GDR. We have no problem with racism. But we do have some racists here. But they are all East Germans and it's them, it's not us.' So this idea of othering East Germany and creating this area or this room where we put all, we delegate all the characteristics we don't want to have, or we don't want to admit to into this little box, East Germany….it's a tool to not talk about racism in Germany. Which means West Germany. And also East Germany, we don't talk about it.”—Participant 13

#### Homogenization of racialized populations/invisibility and erasure

Participants described how these narratives have been and continue to be operationalized in myriad ways throughout German society and not just at the personal level, but also reinforced and reproduced at structural levels through constructs used in administrative statistical reporting, policies, practices and norms within governmental and non-governmental social service agencies, housing markets, and educational and employment systems. The potential for negative impacts are great as these systems structure opportunities for living in healthy environments.

The phrase “of migration background” is a perfect example of homogenization and erasure, key features of racism and colonial thought with important public and environmental health implications. Uniformly, participants stated that the phrase “of migration background” and it use in governmental statistics is problematic.

“I think the term [migration background] is actually very...for black people.. problematic because we‘re all, again, we're lumped in one big bowl with everybody else. What does this mean? A migration background of a Polish person is different than of an African person or an Afro Caribbean person, because we experienced racism.”—Participant 4

“Migration background” is an official statistical category used by German governmental agencies and research institutes for health surveys and health monitoring (118). The German Federal Statistics Office classifies a person who was born as a non-German citizen or born to at least one parent without German citizenship as having a ‘migration background' (119). Despite the official census definition, public health researchers also use the demographic information of grandparents to establish the category of ‘migration background' [see Brinkmann et al. (19)].

Participant 3 argued that the phrase “*of migration background”*, often used as a euphemism for race, lacks any real meaning.

“…race is not a German problem…but migration is. That is the argument behind it. They focus so heavily on migration research or research on migration background, because they have this of course the guest worker family, 4th generation now… But it doesn't mean even you migrated now, it means like you have migration history…which doesn't say anything about your positionality or reality. And it also includes white migrated people from the eastern parts. So that's why they can always say that migrant background people are not doing so bad. Because they count in the non-racialized migrants.”—Participant 3

Participant 3 also pointed out that this terminology “has been criticized a lot for the last two decades”. However the current solution is no better.

“So now we are in the phase of don't use it without being critical about it. But now they frame it, especially in the context of public health and epidemiology, as migration history, if your family has migration history. Which is replacing the same term. Remember we don't have race categories here. We do not use the word race. The German word …*die Rasse*….is so problematic. Germany missed the point where they could translate the biological understanding of this word into the social concept….which it is…because there is no biological basis. There is no discourse about this here.”—Participant 3

Continued use of “migrant background” or “migration history” in health and other official statistics only serves to mask the lived experience of racialized populations. This creates data gaps resulting in these communities being overlooked when governmental and non-governmental organization are addressing and implementing policies to reduce exposures to environmental hazards or prevent harms from climate change.

“One of the organizations that I know is, Friends of the Earth-Germany where they will say to some of the Black people who have been working in that organization, ‘what are you doing, all of these anti racism and anti-colonialism things?' They [FOE] are afraid to lose their members, but I think more than that, they themselves are very heavily invested in seeing environment as something that is, can be looked at or worked on outside of social justice. And there's not a single organization that has, to my knowledge, really embraced this idea [that environmental issues are linked to colonialism and social justice].”—Participant 12

#### Intersectional/intersectoral actualization of racism

It is interesting that the concept “migration background” may have originated during the guest worker programs in 1950s and 1960s in Germany. One participant described the historical segregation of social services in Germany as a structural driver of inequities and disparate treatment of minorities in housing, education and health care. Prior to 1999, social service agencies were segregated in Germany (120). After World War II, Germany recruited millions of so-called “Guest workers”, first from Italy, then from other southern European countries (Greece, Spain, Yugoslavia), and later from Turkey. As these workers sought social services and social support, they were assigned to specific social service providers who were of the same religion and community. But as noted by Participants 11 and 14, this historical segregation likely contributed to cultural essentialism that may explain maltreatment of immigrants, Muslim families and Afro-diasporic people by governmental agencies and social services today.

”A single person, or sometimes like two people at the *Jugendamt* (youth social services agency) can make decisions that affect families for a lifetime (e.g., child endangerment report), and they obviously are informed by their own belief systems, which is understandable, but I feel like there's not enough tutoring on critical whiteness or multicultural perspectives.”—Participant 14, social worker with an NGO in Berlin

These stereotypes then pervade current social and health care service agencies. Participant 11 pointed to ongoing perception that a “traditional” Muslim person does not have a very strong educational background and “approach issues health and self-care more from religious understandings.”

“Institutions who are supporting parents of autistic children approach Muslim families in a different way than they do, families from the so called majority society. There is this belief that Muslim families/parents are not informed enough about the illnesses of their children and do not care enough about how to help them with the means of the health system. They are infantilized (the parents).”—Participant 11

Another participant talked about a stereotype in Germany called *Morbus Mediterraneus* which is a racist idea that women of African descent and Middle Eastern Women exaggerate their pain. This concept is used to justify denial of health care.

“It makes it horrible because you‘re not, you're not listened to, you‘re not seen, you're not taken care of. I hear it more and more. It matters. The more African you get, the more Muslim you look, the greater the level of neglect [by the health care system].”—Participant 7

Participants also described how the disparate treatment by race (and language status) is “baked-into” the norms in the educational system and how this has cascading effects. Black children or children perceived as “of migrant background” are often tracked to the lower path for secondary education in Germany [either the *Hauptschule* (lowest) and *Realschule* (middle)] that does not lead to university. This “tracking” starts when the child is 10 years old. The German educational system replicates and reinforces a social hierarchy, even by race.

“Teachers usually try to put the [migrant] children in like what they call a special class. Because they say that these children need help, like, for example, German, because the parents don't speak German at home, so they would say, ‘oh, they need extra help in school'. This is what they call, like, a *Förderklasse*. And when they start there, they continue on in a *Förderklasse*. They say, ‘okay, this is just to help them, just to help them', and so on. The parents think, oh, this is good. But this is also very negative for them because they wouldn't get the recommendation to go to a gymnasium.” -Participant 4

And even if a child makes it to *Gymnasium*, the highest of the three main types of German secondary schools that prepares students for university level education if they choose to continue, Participants described additional barriers.

“Even if you come from a racialized lower socioeconomic status, family or background, and you get to this higher education track, it's very difficult to stay there because of racism and also because of socioeconomic status, because then the [higher socio-economic status] parents, they can pay tutors.” -Participant 3

“Teachers speak of schools with a very high percentage of students, pupils with a migration background, and they don't expect success. And this is racializing, socioeconomic disadvantage. You have this in the education system, very clear in Germany, the racialization of the disadvantage.”—Participant 11

Participant 1 noted that “because of intersectionality people from weak social classes are at the same time people of color”. But even with a college education, participants described instances where people have been denied a job because the applicant had a Turkish name or African sounding name. Recent findings by the German Center for Integration and Migration Research (DZIM) show correlation between racism and the risk of poverty, especially for Muslim men (121).

The housing sector was also identified by participants as a point where individuals have unchecked power to discriminate. One participant described housing as “one of the most obvious racial disparities” that occurs despite the existence of anti-discrimination law in Germany. This participant related stories where an applicant either sends in a copy of photo ID or comes in person to view the apartment after the rental application was accepted and the landlord, based on the racialized appearance of the applicant, rescinds the offer with no more than ‘sorry, I don't have an apartment for you'. The law has limitations.

“In a law setting, you [must] prove you were disadvantaged because of race or gender. So a lot of people [say] we don't need the term racism or don't need to understand racialization. We just need to understand discrimination. But discrimination is already so invisible, especially on a structural institutional level [like] who's in a structural function or has structural power, which are like the people who rent their flats. And then if they have racist ideas of certain groups, they don't give you the flat. So, how do you prove that they did that because of that. There's also a lot in the Afrozensus data where people report they got accepted for a flat before it was obvious they were Black.” -Participant 3

### Pathways toward solutions for addressing racial inequities in health, healthcare and promoting environmental justice in Germany

#### Addressing structures and improving governmental initiatives

When discussing solutions, participants focused more on the institutional and structural than individual aspects (e.g., racial animus directed at individuals). For example there was strong endorsement by participants on the need to improve not only representation of racialized minorities in positions of power and decision making inside government, higher education and health care institutions, and civil society organizations, etc., but also the policies, practices and norms of these organizations.

“The decision makers mostly are people not directly concerned by the issues [racism]. So, when they decide, they decide with their minds and so on. So that's why I think it's very, very important that people like me work in those structures because, of course, we'll be giving advices and so on.” -Participant 2

One participant described how their social service organization intentionally structures their hiring and operational processes to value and reflect the lived experiences of the minoritized communities they serve, which could be a model for others.

“People with academic requirements and people who have the cultural interpersonal experience work in co-teams. Which is very rare that your experience, your cultural experience is accounted for on a similar level as an academic degree, which opens chances to our coworkers to first of all, give something back and then to get an equal pay compared to people with an academic degree who do the same job.”—Participant 14

While Participants named a few governmental initiatives to address racism, they noted that successful solutions require these governmental bodies to actually follow-through on recommendations, take action and set up processes for accountability. One participant reflected on her disappointment with the Berlin Senate ignoring recommendations she and others were invited to develop several years ago regarding climate impacts, infrastructure and racial discrimination. Her recommendations had referenced the Berlin Senate's Environmental Justice Atlas.

“They call it Environmental Justice Atlas to see how pollution is distributed unequally. And they also have included economic resources or income in their analysis. It's clear there's environmental injustice in Berlin. But I have not read about, heard of any policies or anything resulting from that [report] or even any measures and I try to follow their work.” -Particpant 12

But even with the extraordinary government initiatives to address racism recently launched in Germany [e.g., National Discrimination and Racism Monitor (NaDiRa)], another participant also expressed doubt about follow-up.

“I'm a bit skeptical when it comes to this topic. We had a project on racism in medical curriculum…things like how textbooks are written, how stereotypes are furthered through teaching materials and things like this. Our recommendations are to revise these textbooks and to think about how things are being taught. The recommendations were published in this report and given to the German Government. We'll see if anything comes of it.”—Participant 7

#### Engaging civil society

Civil society has a role to play in addressing structural inequities in health and environment, especially community-based organizations operated by and serving minoritized communities in Germany.

“The good work is only done, in my opinion, or mostly done by really small initiatives [by community based groups] and not seen by academic institutes and by scientists. The higher it goes on the ladder and the more funding they have, I feel like less they address racism and that is a problem in itself.”—Participant13

Yet, these community-based organizations are not regularly being invited to participate in relevant policy debates or program planning. As Participant 12 noted, there is a “lack of transparency in policy making process” and “ lack of understanding on how to be inclusive” in Germany compared to other EU countries. Other participants endorsed these observations, as reflected in their ideas for solutions to create structures to include marginalized communities in environmental and health policy development at all levels of government and not just consultation after decisions have been made. In other words, German policy makers need to start seeing civil society groups “as equals”.

“I think that community based organizations should be the first stop where government organizations can connect. For example, like that city that had a *Gesundheitkiosk* (health kiosk or retail health clinic located in disadvantaged neighborhoods) to provide information on how to access health care, help you find an appointment…but I don't know any Black person who knows where this kiosk is. If it is run by a migrant organization or by migrant people who know these issues, the community would be more informed.”—Participant 6, Health Care Provider/Physiotherapist in Köln

“There should be much more collaboration between, not just with the politicians, but [with] the ones who are on the staff [of government social service agencies]. What I wish for is that these state institutions or political institutions come and ask us for support…come to us and [and say] they want to learn from us.”—Participant 8

#### Overcoming ignorance/raising awareness

Participants all shared the perspective that in order to break the structural and systemic manifestation of racism, knowledge and awareness about racism, the underlying narrative/values that animate it, and how it works must be increased, especially in German universities, academic and research institutions, main-stream media and among people in power, so that counter measures can be developed and implemented. Otherwise the harms experienced by minoritized groups in Germany will continue to be normalized.

“We need to bring more knowledge about racism to the mainstream or to the white society and to academic institutions and they have to act more political in a way because they are like, ‘we are neutral', [but] you can't be. They have to use their role of power to also strengthen the idea that we need to talk about racism much more in Germany, and then we need to spread the knowledge about racism much more in Germany.”- Participant 13

Several Participants affiliated with research-based organizations noted that academic (especially applied sciences universities) and research institutions can help increase awareness of structural racism and its impacts on health and environmental conditions in communities. But Participants emphasized that this requires the adoption of critical pedagogy, improvements to allied health sciences and medical curricula, better conceptualization of theoretical bases for research on racism, health and environment, effective research translation, and support for community-based participatory action research approaches.

‘As an educator, I would say in all institutions that train and educate people who take over responsibility for health issues in society, that racism, critical approaches have to be a part of the education of the training… but also the knowledge about the damaging factors of racism in the healthcare system.” -Participant 11

“[Racism] needs to be, at some point, mandated into, the curriculum and the text books..so that people are educated and really think about race in medicine. It is really important why we need to be moving away from this genetic and biological discussions. For example, certain things are taught, but without having the back story, or kind of, so people jump to conclusions. And then it's just because they weren't taught properly.”—Participant 7

#### Improving academic research

In addition to improving pedagogy, participants also highlighted the need for strengthening the conceptualization of racism by German public health researchers. The current focus on economic factors solely as drivers of environmental health disparities “speaks to the little concern and recognition that race and racism are given in Germany” and that “[racism] is completely excluded from environmental justice as a concept [in research] and in practice in Germany” (Participant 12). Yet, while there is increased attention to racism and health as an intellectual question, Participant 3 commented that there is “very deep denial” and “epistemological ignorance” in Germany because the research questions are still being framed in the context of migration and whether racism exists or not at the individual level as interpersonal racism and *alltäglicher Rassismus* (everyday racism) in Germany. Rather, academic research should focus on the rules, policies, norms of operations and not just the personal interactions; this means identifying the decision points, the points of power and their racialized impacts on health and environmental outcomes. Therefore critical research, with strong theoretical bases drawing from racial capitalism, Black feminism, history and philosophy, are needed in Germany to understand and overcome structural mechanisms causing environmental, health and structural inequities as illustrated by these quotes from Participant 3.

“it is not enough to focus on racial identities. I wouldn't even call it racial identities, because of the social positioning or social category. But.. we need to move away from just linking race to whatever. And that's why I am trying to focus my research on racialization processes. I don't want to discuss with people if they use terms *rasse* or migration, instead I want to figure out the processes of racialization.”—Participant 3

“If we see that racism and socioeconomic status are linked, then we have to talk also about colonialism. But we don't want to talk about colonialism. So if you deny colonialism, you're not linking racism, class, or capitalism, or socioeconomic positions, history of … stereotypes in text books (especially in medical training) and expand environmental and public health curricula to include examining social justice movements led by marginalized groups from current and prior periods.” -Participant 3

However, it's not enough for “academics to work with other academics” according to Participant 7 and several others. “All these beautiful intellectual theories and academic knowledge” being produced by DIZM, or even by decolonial initiatives like Decolonize Berlin, need to be translated back “into the practice” (Participant 3) and “benefit families and communities” (Participant 8) impacted by the legacies of colonialism and ongoing structural inequities. Several participants recommended that research needs to be grounded in the experiences of minoritized people, because “just the numbers doesn't tell you what's going on” (Participant 3).

To better ground research on environment and health inequities, “knowledge needs to be brought from bottom up” (Participant 13) and that researchers need to listen to the people who are “actually affected by the institutions, because oftentimes people from the highly ranked positions have little to no exchange with the actual parties they make decisions about” (Participant 14). We discussed how community-based and community-engaged participatory action research approaches (122, 123), well known in the U.S. and the U.K., could help advance the priorities of communities and engage community partners in the planning and implementation of research, but are not widely practiced or valued in Germany.

#### Challenges for the future

Even as Participants articulated a number of solution ideas (e.g., increasing representation in positions of power, strengthening academic research efforts, etc.) doubts remained about real change. For one, structural racism advantages some groups over others by definition and Participant 13 expressed doubt that people will “voluntarily giving up their privileges here in Germany” just because they are more aware of racism's effects. Hence the need to focus on eliminating structures of racism at the same time. Other reasons for the doubts shared by participants lay in the contradictions stemming from the rise of anti-immigrant and anti-migrant sentiments and growing power of the right-wing party, the Alternative für Deutschland (AfD).

“We have provided a lot of knowledge about racism through the national anti-discrimination and racism monitor (DZIM). We have initiatives like the Afrozensus, which are supported also by state institutions. Also by foundations that have an awareness toward these issues. But we have contradictory developments in Germany. We have on the one hand this awareness, which is much higher than it was 10 years ago in the governmental institutions. And in also in, in the civil society, in the big foundations. And on the other hand, you have voters who vote for a party like the AfD.” -Participant 11

Participant 12 noted that there is growing push bask against the discussions around racism in Germany. The impact on environmental topics or environmental racism “will be less capacities for everyone working against racism, less discourse and also resulting in less policy space to raise these issues because there will be less interest or care.” Against this backdrop of growing right-wing political power and sentiment, some Participants admitted that they, as researchers, and German society as a whole, lack a narrative to effectively push back against right wing extremist sentiments.

“German federal government researchers on these topics [racism] are not equipped to communicate the important work they are doing and to stand up to backlash. And instead, they are afraid and so they just put their head down. There is this paralysis. They don't know what to do, and so then they don't do anything.”—Participant 12

“Then I ask myself what kind of democratic background do we have? What kind of groundings in democracy do we have? Did we fail so much to give people a sense of how precious pluralism and democracy is?”—Participant 11

“And I feel like now it's rather a symptom of what is brewing all the time because we didn't do the work for *Nie Wieder*, Never Again.”—Participant 13

In the near term, the hopeful path forward lies in solidarity and doing the hard work identifying, challenging, and changing the systems, policies, attitudes, and behaviors that perpetuate racial inequities because “racism is global” (Participant 9) and it may take “two generations to see change” (Participant 2).

“We should find solidarity with other communities and groups to oppose what is coming from the right.”—Participant 13

## Discussion

Qualitative interviews with Black German researchers, educators, and health care and social service providers revealed contradictory developments regarding efforts to address racism and the impact on environmental conditions and health. It's not a question of whether racism exists in Germany. Of course it exists, especially anti-Black racism (14, 124). Neither is it a question whether people are talking about racism or not. Of course people are talking about racism in Germany as evidenced by numerous reports (16, 17, 20, 42, 75) and governmental initiatives (72, 74, 75, 77). Reflections from interview participants, however, show that the topics that have yet to be “put on the agenda” in mainstream German society generally, and among academic health sciences institutions in particular, are the underlying values and norms that support racial ideology and hierarchies, the structural mechanisms of racism in Germany, the health and environmental consequences, and solutions that don't just individualize racism by putting the responsibility on the victim of racism (e.g., filing discrimination claims; seeking mental health providers).

To my knowledge this is the first analysis of qualitative interviews with Afro-diasporic people in Germany on the manifestations and root causes of environmental health inequities. These interviews highlighted ongoing environmental health inequities impacting Black and racialized populations in Germany, however made invisible due to the omission of these experiences from governmental health statistics and data collection mechanisms. Consistent with the Needham framework on structural racism and health (63), participants pointed to the ways racism is structured in Germany, tracing how racialized and racially hierarchical values and beliefs (e.g., persons of color are “strangers” and not real Germans; migrants are dirty and only Germans are clean) become actualized through discriminatory policies and practices (e.g., use of “migration background” as an official statistical category) embedded in social institutions such as the government, the economy, the education and the healthcare systems, which lead to racialized outcomes in health and environmental conditions. Participants painted a complex picture of racism operating in Germany, confirming a long standing lack of dealing with German national history and denial of racism (64, 125). Defeating Nazi socialism didn‘t solve racism (91, 117, 126–129). Insights from participants suggest that German federal government initiatives on racism will likely not yield tangible results if they are not coupled with structural change, engaging civil society (especially groups led by and/or serve racialized minority communities) and translating research on racism into practical applications, including the development of counter narratives to “main German culture”, right-wing and anti-immigrant sentiment. Thus, to move forward, a more structural conceptualization of racism in Germany, like Needham's definition and framework, is needed to better inform public policy and environmental health research. To help identify, reimagine, and redesign social structures that sustain structural racism, potential measures corresponding to the multidimensional features of institutional, structural and systemic racism that could be adopted for research in Germany are illustrated in Supplementary Table S3.

This study revealed several implications for environmental health research and promotion in Germany vis-à-vis the EU Research Agenda for the Environment, Climate and Health 2021–2030 (The EU Research Agenda). The lack of data on racialized populations as part of regular health monitoring or other census data in Germany is a significant barrier to realizing the EU goals on environmental injustice and equity. Participants described several conventional environmental health problems likely disproportionately impacting racialized minority populations in Germany, such as air pollution and substandard housing, but also named racial profiling and surveillance by police as an environmental stressor. This expanded view of environmental stressors, is worthy of further investigation. However, the ability to fully understand the extent of any environmental exposure and the impact on the health of racialized populations in Germany is obscured because it requires data (124). The use of “migrant background” or “migration history” as a construct for public health statistics is inadequate. However, the Afrozensus (14) and surveys by NaDiRa (130) show that self-reported racial identity and lived experiences information can be collected and these approaches could be a model for governmental administration responsible for environmental and health statistics as well as for primary research on environmental health. While the Afrozensus and the DeZIM Institute's report *Rassismus und seine Symptome* have put a national spotlight on the relationship between “everyday racism” and health in Germany, the hyper focus on mental health effects and increasing the availability of counseling as the solution may only serve to individualize these experiences. Structural causes may be ignored. Efforts by governmental or academic institutions to assess how institutional and structural racism affects other health outcomes related to environmental exposures such as pre-mature mortality (131), accelerated aging (132), cancer outcomes (133, 134), maternal morbidity (135) and mortality (136), pre-term birth (137) and low birth weight (138) infant mortality (139), which have been documented in the scientific literature in the U.S., are not yet on the horizon as topics in Germany, but should be.

The EU Research Agenda also calls for creating new knowledge on societal and social structures and mechanisms, including embedded values and narratives, leading to differential exposure to environmental and social stressors. Participants in my study theorized that the paucity of empirical studies on racism, health and the environment in Germany is also driven by the dominant conceptualization or narrative in German society that racism is linked to migration. In other words, as historian Fatima El-Tayeb argues, racism is often framed as being brought with the arrival of “migrants” and not discussed as something already present in the dominant culture (140–142). This problematic narrative brews epistemological ignorance/”colonial amnesia” and lack of critical thinking and awareness at the individual level (by whites) of history and how biases are reproduced. As participants described, the society then is left vulnerable and unprepared to counter right-wing politics and extremism, which is on the rise in Germany, as well as to confront structural racism and environmental injustice. In order for the EU research Agenda on environmental health and anti-racism initiatives in Germany to yield meaningful results, both need to be in conversation with each other and more aligned to uncover and address the systems, the processes of power and racialization that are operating in German society.

Lastly, environmental justice topics in Germany tend to be confined to elite spaces of academic research and not grassroots, community- driven social -justice efforts. Through these interviews, it's clear there is a lack of uplifting and building upon social justice work by minority communities within Germany. For example white German researchers on environmental justice and health disparities often cite U.S. and U.K. research but rarely mention Germany's own Black scholars on race (e.g., Fatima El-Tayeb), Black feminist scholars and activists-intellectuals (e.g., Katharina Oguntoye, Jasmin Eding, May Ayim) or black and minority serving organizations and initiatives (e.g., Each One Teach One, ADEFRA, Kotti & Co, Black in Medicine Netzwerk Schwarzer Mediziner^*^innen, Initiative Schwarze Menschen in Deutschland, etc.). Increasing participatory research with minoritized and impacted communities to address environmental injustices is needed, especially on topics of housing justice, air quality, climate impacts, children's environmental health and mental health.

This study has some limitations. The technique of purposive sampling was used to recruit individuals who self-identified as Afro-German or worked in organizations focused on addressing the health and environmental challenges faced by minoritized populations in Germany. However some perspectives and geographical representation may be underrepresented. It is important to note that the narratives examined here are specific to the given interview context and time period when these interviews were given. By conducting the interviews in English, nuances unique to the German language may not have been captured. While 50% of interview participants self-identified as Black, and 57% worked directly with racialized communities as part of their professional responsibilities with health care, community-based and other non-governmental organizations, these individuals represented what I call “grass tops”. Further work should attempt to reach a broader sample of participants representing the “grass roots” such as racial minority residents at a neighborhood level. Collaborating with German and European Afro-diasporic focused organizations such as Each One Teach One, *Initiative Schwarze Menschen* in Deutschland and Equinox Initiative for Racial Justice on environmental health equity would be an ideal next step. Despite the limitations, findings from this study affirm previous examinations of racial discrimination and anti-immigrant narratives in Germany. While building on prior work, the unique contribution of this present study is in-depth exploration of environmental health inequities, their structural causes and solutions from the perspectives of racialized individuals in Germany. Results provide direction for improving the conduct of public health research, the development of policies, and ultimately the outcomes under German government initiatives on racism.

## Conclusion

Since 2020 the discussion about racism has been booming in Germany, led by a number of federal government initiatives, but with very little attention to environmental health consequences or on the underlying historical, structural or cultural narrative root causes. This disconnect is readily described by anti-racism researchers, community-based advocates and educators knowledgeable about environmental justice, racism and public health who identify as Black or Afro-German and/or work on behalf of racialized communities in Germany. This study provides in-depth exploration of environmental health, their structural causes and solutions from the perspectives of racialized individuals in Germany. Environmental health inequity concerns ranged from air pollution to spatial segregation in Germany, but the extent of these harms is unknown due to lack of data. Interview participants identified pathways toward more effective research and policy initiatives on racism, environmental justice, community health and environmental heath equity in Germany including: collecting better data that is informed by structural theories of racism/racialization and power; supporting more community-based participatory action research; improving accountability of governmental agencies; engaging with existing civil society and non-governmental organizations that serve and advocate on behalf of minoritized communities; and building upon past and present progressive social movements. With the increasing popularity of right-wing and anti-immigrant sentiment in Germany, deep work is needed to address history and national cultural narratives to foster a more inclusive, pluralistic and democratic society. Otherwise, all of the recent government-led anti-racism initiatives risk failure. Structural racism and its relationship to health and environmental inequities is a global phenomenon. Engaging in more critical international comparative dialogue on racial practices, anti-racism research and lessons learned from legal, cultural and moral initiatives to address racism is needed.

## Data Availability

The original contributions presented in the study are included in the article/Supplementary material, further inquiries can be directed to the corresponding author.
