# Supplementary material for: “Remember, we don't have race categories here”: contradictions and reflections on racism, environment, and health from an interview study among Black German researchers, educators, and care providers
Source: Front Public Health. 2025 Dec 19;13:1658436. doi: 10.3389/fpubh.2025.1658436 (PMC12757308; doi:10.3389/fpubh.2025.1658436)
Supplement: Supplementary file 2 [file Table_2.docx]

Table S2. Themes and their features under RQ1: What are examples of health and environmental inequities disproportionately affecting racially minoritized communities in Germany?

| Theme | Description  (what it represents) | Cases  (# of participants) | Counts  (# of empirical indicators) | Example Empirical Indicator (Evidence) |
| --- | --- | --- | --- | --- |
| Environmental Exposures and the Built Environment | This depicts wide range of environmental exposures, low housing quality of immigrants, Roma and Sinti communities, and other racialized people, and spatial segregation that negatively impact minoritized populations in Germany | 9 | 33 | “Without knowing or having any official data where exactly most black people live in Berlin but based on what is community knowledge, where we will find each other, we can deduct from the state (Berlin EJ Atlas on environmental pollution) that black people in Berlin are disproportionately affected by environmental issues…pollution.”  (P12)  “But regarding housing there are some difficulties that families face because some of the families we serve live in [Gemeinschaftsunterkünften](https://de.wikipedia.org/wiki/Gemeinschaftsunterkunft), (shared accommodation)…refugee camps, but it is like old buildings are rebuilt to host refugees, like old hospitals.” P14  “But where they [refugees] are placed [is] in very dirty areas and very loud areas and so on, and they place them in very rural areas where you have no infrastructure. Then one could ask ok maybe there is less air pollution, but it is more danger because of your physical safety or mental health. “ P3 |
| Police Interactions and Spatial Segregation | This indicates participants including mistreatment and killing of minoritized people by the police in Germany as an environmental health inequity | 3 | 3 | “So there was a case, in the year 2000 and everything was showing clear it was a murder by the police.”  (P2) |
| Health and Health Care | This theme encompasses all references made to disparities in health outcomes and access to health care in general and to specific health outcomes such as mental health and COVID-19 | 11 | 49 | “Like, when it comes to mental health, we're beginning to understand that racism impacts mental health and at the same time it's really hard to find a therapist.”  (P7) |

Table S3. Themes and their features under RQ2: What are the historical, cultural and structural causal mechanisms for these inequities?

| Theme | Description  (what it represents) | Cases  (# of participants) | Counts  (# of empirical indicators) | Example Empirical Indicator (Evidence) |
| --- | --- | --- | --- | --- |
| White German Narratives About German National Identity | This theme encompasses the experiences and observations by participants on how minoritized groups are othered by White Germans and the narratives mainstream Germans tell themselves about their national history | 11 | 77 | “...there's still this idea in Germany that a real German is white. There's always an idea of, you have to be a foreigner [a person of color though born in Germany], you are different, you are like Fremde, you are alien to the society, you are not a real part of the society, and you will never be, because you are not white. In sociology, we would call the racism we have in Germany Blut und Boden Racism (blood and soil racism).”  (P13) |
| Right Wing Politics | This refers to the growing power of right wing politics in Germany and the challenges it presents to confronting racism and environmental injustice. | 7 | 14 | If you have all of the Eastern German Länder(Eastern German states), 32 percent people who would opt, would vote for AfD (Alternative for Germany right-wing populist political party). The party is much more right wing than the same party in the western part of Germany. Then I ask myself what kind of democratic background do we have? What kind of groundings in democracy do we have? Did we fail so much to give people a sense of how precious pluralism and democracy is?”  (P11)  “The far right is also claiming more environmental even starting to claim climate issues for themselves…more in the sense of protecting the homeland (from foreigners).”  (P12) |
| Denial of History | This refers to the many ways participants have observed White Germans deny history and deflect from actually dealing with the country’s history. | 10 | 37 | “Children are not taught history – doesn’t matter if the person is 6 or 66.. about the colonial past of Germany. They say it was short and has not impact on the present. This is the narrative.” P1  “Once you start, once you start speaking, talking about colonialism and what they used to do is to compare and say the French colonialism was longer and so on. That's what we do in Germany. It was long and it was more brutal. No, we did not do anything. We just brought a piece and so on. It's a problem.” P2 |
| Homogenization of Racialized Populations/ Invisibility and Erasure | This depicts a wide range of efforts, consciously and unconsciously, by German society to erase the presence, contribution, and suffering of Afro German, persons of color and minoritized communities. | 14 | 95 | “I think the term [migration background] is actually very...for black people.. problematic because we're all, again, we're lumped in one big bowl with everybody else. What does this mean? A migration background of a Polish person is different than of an African person or an Afro Caribbean person, because we experienced racism.”  (P4)  “Because you're not, you're not listened to, you're not seen, you're not taken care of, you know. Yeah, I hear it more and more, and it, I think it, yeah, it matters, the more, the more African you get, the more, you know, Muslim you look, the greater the level of neglect.”  (P7) |
| Intersectional/Intersectoral Actualization of Racism | This indicates references made by participants to the interconnectedness between racism in one sector (e.g. education, housing, employment) and disadvantage and discrimination in another sector of society. | 13 | 65 | “Teachers speak of schools with a very high percentage of students, pupils with a migration background, and they don't expect success. And this is racializing socioeconomic disadvantage. You have this in the education system, very clear in Germany, the racialization of the disadvantage.” |

Table S4. Themes and their features under RQ3: What are potential solutions for addressing racial inequities in health, healthcare and promoting environmental justice in Germany? What progress has been made, and what challenges remain?

| Theme | Description  (what it represents) | Cases  (# of participants) | Counts  (# of empirical indicators) | Example Empirical Indicator (Evidence) |
| --- | --- | --- | --- | --- |
| Addressing Structures and Improving Governmental Initiatives | This theme references participants’ perspectives on the need for governmental agencies and other organizations to focus actions at systems level change in order to effectively address racialize inequities and to counter how governmental agencies and non-governmental agencies institutionalize racism through both action and inaction | 14 | 47 | “And that's what I also sense.. a lot within this community social movement organizing or building community institutions where you then sit there and you're like, okay, we are talking a lot about intersections and so on, but structurally we're not doing something about it.”(P3)  “There's this publication, Environmental Justice Atlas, in Berlin, and it shows how pollution is distributed unequally. But I have not read about or heard of any policies or anything resulting from that or even any measures. I'm not aware of any measures and I try to follow their work.”  (P12) |
| Engaging Civil Society | This represents participants’ views on the valuable role community-based organization can play in addressing social injustices and in putting pressure on mainstream organizations to change practices but also the need for NGOs to organize, coordinate and collaborate | 9 | 32 | “A solution could be to just cause more overlap [coverage, engage with each other, coordination] to just first of all, maybe to develop more [community-based service] organizations like ours…with a specific target group who fights against injustices or fights against disadvantages or tries to fill in the gap.”  (P14)  “I think what is missing most times in Germany is the connection to people so there needs to be more support for community-based organizations. They have been speaking about this for years, that Black organizations receive far less funding than other organizations.” (P4) |
| Overcoming Ignorance/Raising Awareness | This theme encompasses overarching recommendations to educate the public, heath care provides, employers, employees, broader society, etc., about structural racism in Germany. | 10 | 45 | “As an educator, I would say in all institutions that train and educate people who take over responsibility for health issues in society, that racism, critical approaches have to be a part of the education of the training.” |
| Improving Academic Research | This refers to participants recognizing the special role institutions of higher education play in Germany in perpetuating racist ideas or reducing the framing of racism as only at the individual level and therefore should do more to combat racism through their pedagogy and research | 8 | 30 | “Need to bring more knowledge about racism to academic institutions and they have to act more political in a way because they are like, ‘we are neutral’. They have to use their role of power to also strengthen the idea that we need to talk about racism much more in Germany, and then we need to spread the knowledge about racism much more in Germany.”  (P13)  “Especially no one is talking about structural and institutional racism and everyone is talking about interpersonal racism and alltäglicher rassismus ( everyday racism). Where I am like, what exactly is that. It happens every day to you because it is set in the structure, in the culture in the behavior and so on.” (P3) |
| Challenges for the Future | This theme captures both the hopes and fears about progress as expressed by participants | 6 | 19 | “So, I'm not really hopeful that we will see the change. Maybe two generations after us. But, uh, yeah, the change has started anyway. “  (P2) |
