# Supplementary material for: “Remember, we don't have race categories here”: contradictions and reflections on racism, environment, and health from an interview study among Black German researchers, educators, and care providers
Source: Front Public Health. 2025 Dec 19;13:1658436. doi: 10.3389/fpubh.2025.1658436 (PMC12757308; doi:10.3389/fpubh.2025.1658436)
Supplement: Supplementary file 3 [file Table_3.docx]

**Table S3 Features of Institutional, Structural and Systematic Racism and Potential Corresponding Measures**

| Needham Conceptual Framework | Features of Institutional, Structural and Systemic Racism | Illustrative Example Measures |
| --- | --- | --- |
| Racialization | Citizenship laws | Who is excluded and not considered a full citizen with access to power, resources, opportunities, etc. |
|  | State control and surveillance | Racial profiling, subjecting members of certain population groups to random search, presentation of papers, etc. |
|  | Geography and spatial segregation | Various forms of segregation, limiting minority groups access to important social resources |
| Ideological Racism | Devaluation of human worth | Beliefs about hierarchies of human value and deservingness (e.g., anti-Black, anti-Muslim, anti-immigrant sentiments)  Homogenization of heterogenous groups (e.g. “of migration background) |
|  | Historical roots and amnesia | Ignores history and treats it as unimportant and having no bearing on contemporary society  Erases the recognition  of historical processes that would link contemporary racist institutional policies and practices to explicit racial intent. |
|  | Popular and political discourse and images | Who is worthy of symbolic citizenship based on racialized group membership (e.g., “of migration background”)  Which groups’ histories and cultures are included in that of the nation through media portray, celebrations, public art and educational curricula.  Stereotypes and discourse by dominant group of who is “good” and “bad” that essentialize difference, inferiority and superiority |
| Actualized Racism | Institutional manifestations and perceived neutrality | Specific laws, policies and practices of various institutional and organization domains such as the labor market, health care, housing, education that may appear race-neutral, when in reality,  they originate from an underlying intent to surveil, dispossess, and control, certain  racialized groups  Perceived neutrality is  deeply tied to ahistoricism |
|  | Ubiquitous adaption | How configurations of institutional and cultural practices change to adapt to local sociopolitical norms across place and time to maintain racial order.  Where racial progress in one area (e.g., politics,  criminal justice, labor market, education, and health care) is met with racial retrogression in another area |
|  | Network structure | Shared cultural norms of  institutions and how they are maintained across both place and time and thus maintain racial inequality |
| Racial Inequity | Disparities in outcomes | Measures of disparities by race in income, employment, housing quality, environmental exposures, health outcomes, etc. |

Note: Content adapted from Hicken 2021, Lee 2024, Needham 2023, and Weiner 2012
